# Oxidative Debromination and Degradation of Tetrabromo-bisphenol A by a Functionalized Silica-Supported Iron(III)-tetrakis(*p*-sulfonatophenyl)porphyrin Catalyst

**DOI:** 10.3390/molecules18055360

**Published:** 2013-05-10

**Authors:** Qianqian Zhu, Yusuke Mizutani, Shohei Maeno, Masami Fukushima

**Affiliations:** Laboratory of Chemical Resources, Division of Sustainable Resources Engineering, Graduate School of Engineering, Hokkaido University, Sapporo 060-8628, Japan

**Keywords:** iron(III)-porphyrin, humic acid, silica support, catalytic oxidation, tetrabromobisphenol A

## Abstract

Tetrabromobisphenol A (TBBPA), a commonly used brominated flame retardant, also functions as an endocrine disruptor. Thus, the degradation of TBBPA has attracted considerable interest among the scientific community. Iron(III)-porphyrin complexes are generally regarded as “green” catalysts and have been reported to catalyze the efficient degradation and dehalogenation of halogenated phenols in environmental wastewaters. However, they are quickly deactivated due to self-degradation in the presence of an oxygen donor, such as KHSO_5_. In the present study, an iron(III)-tetrakis(*p*-sulfonatophenyl)-porphyrin (FeTPPS) was immobilized on imidazole-modified silica (FeTPPS/IPS) via coordination of the Fe(III) with the nitrogen atom in imidazole to suppress self-degradation and thus enhance the catalyst reusability. The oxidative degradation and debromination of TBBPA and the influence of humic acid (HA), a major component in leachates, on the oxidation of TBBPA was investigated. More than 95% of the TBBPA was degraded in the pH range from 3 to 8 in the absence of HA, while the optimal pH for the reaction was at pH 8 in the presence of HA. Although the rate of degradation was decreased in the presence of HA, over 95% of the TBBPA was degraded within 12 h in the presence of 28 mg-C L^−1^ of HA. At pH 8, the FeTPPS/IPS catalyst could be reused up to 10 times without any detectable loss of activity for TBBPA for degradation and debromination, even in the presence of HA.

## 1. Introduction

Tetrabromobisphenol A (TBBPA), a widely used brominated flame retardant (BFR), is used in the treatment of paper, textiles, plastics, electronic equipment, upholstered furniture and chiefly in epoxy resins that are used in circuit board laminates [1]. The leaching of BFRs, as well as TBBPA, from wastes derived from such materials in landfills is facilitated in the presence of humic acid (HA), which is a major component in landfill leachates [2,3]. Many studies have shown that TBBPA can induce cytotoxicity and hepatotoxicity, and it has the potential to disrupt estrogen signaling [4], therefore, the development of effective methods for removing TBBPA from landfill leachates is an important issue. Methods have been reported for oxidative degradation of TBBPA (e.g., birnessite oxidation [5], photooxidation [6] and permanganate oxidation [7]), but most involve the cleavage of the β-carbon in TBBPA, and not debromination. In addition, the influence of other contaminants, such as HAs, on TBBPA oxidation has not been investigated in detail, even though it is well known that HAs are major components of landfill leachates.

Considerable interest has developed regarding Fe-based biomimetic catalysts, such as iron-porphyrins, because of their ability to mimic cytochrome P450 enzymes that catalyze a variety of oxidation reactions [8,9]. Iron porphyrins can oxidize bromophenols in homogenous solutions [10,11], but these types of catalysts are deactivated due to rapid self-degradation under the highly oxidative conditions and by dimerization via the formation of μ-oxo iron(III) species [12,13,14]. The immobilization of such catalysts on a suitable support has been used to increase their stability towards oxidative self-degradation and to prevent catalyst deactivation caused by dimerization [15,16,17,18,19,20,21,22]. Among the supports used to immobilize metalloporphyrins, inorganic supports such as SiO_2_ are preferred, because of its low cost, availability, mechanical robustness and chemical inertness.

In a previous study, our research group examined the degradation of TBBPA using a homogeneous iron(III)-porphyrin catalytic system. The findings indicated that the oxidation was not efficient and no debromination was observed because the catalyst underwent self-degradation and inhibition by contaminating HA [10]. Although the immobilization of anionic iron(III)-porphyrins on an anion-exchange resin was examined, the catalytic activity for 2,6-dibromophenol oxidation decreased because of anion-exchange reactions between iron(III)-porphyrin and HA, 2,6-dibromophenol or oxidation products derived from it [23]. In addition, the positively charged surface of the anion-exchange resin can also adsorb anionic HA, which results in a decrease in degradation performance. However, nitrogen atoms that are included in the functional groups of the anion-exchange resins can serve as a ligand for coordination with iron(III). If the iron(III) in the anionic porphyrin could be tightly attached to the nitrogen atom on the support by coordination, the surface potentials of the solid catalysts would be changed to negative after complexation. Using such a type of the solid catalyst, the adsorption of anionic concomitants, such as HAs, would be suppressed, thus producing a stabile form of iron(III)-porphyrin catalyst on the support. 

In the present study, an anionic iron(III)-tetrakis(*p*-sulfonatephenyl)porphyrin (FeTPPS) immobilized on silica modified with an imidazole was examined for use as a catalyst for the enhanced degradation and debromination of TBBPA in the presence of HA. In addition, the influence of HA on the rate of TBBPA degradation, debromination and reusability were investigated.

## 2. Results and Discussion

### 2.1. Characterization of FeTPPS / IPS 

The amount of FeTPPS molecules bound to the surface of the 3-(1-imidazolyl)propylcarbamoyl-3′-aminopropylsilica (IPS) was estimated by the change in absorbance at 394 nm of the Soret band in UV-visible absorption spectra. The relative absorption at a wavelength of 394 nm (corresponding to the Soret band of FeTPPS) between a stock solution of FeTPPS and the solution obtained after removing the FeTPPS/IPS was used to determine the concentration of FeTPPS molecules bound to the IPS. The findings indicated that 32.7 μmol of FeTPPS was immobilized on 1 g of IPS.

**Figure 1:**
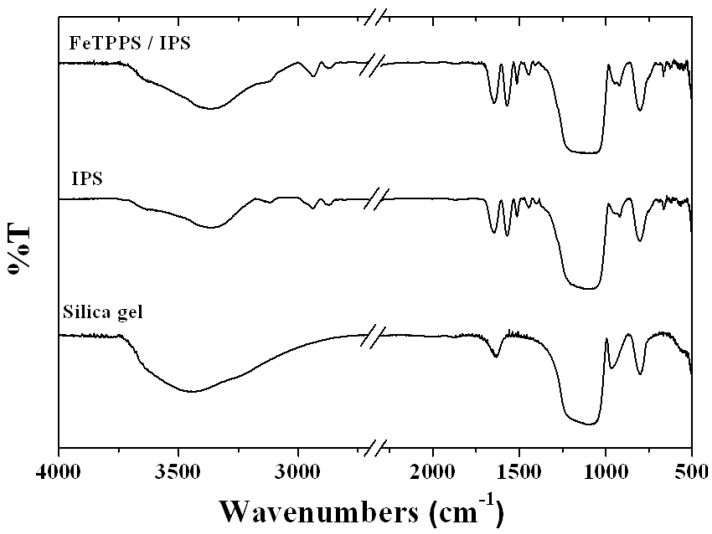
FT-IR spectra of silica gel, IPS and FeTPPS / IPS with KBr pellet.

FT-IR spectra of silica, IPS and FeTPPS/IPS are shown in Figure 1. The FT-IR spectrum of IPS contained characteristic vibration bands in the 2800–3000 cm^−1^ region, corresponding to symmetrical and asymmetrical C-H stretching vibrations. The absorbance in the 1400–1600 cm^−1^ region is assigned to C=C, C=N ring stretching (skeletal bands) as well as the C=O stretching vibration which was observed in the FT-IR spectra of IPS and FeTPPS/IPS.

**Figure 2:**
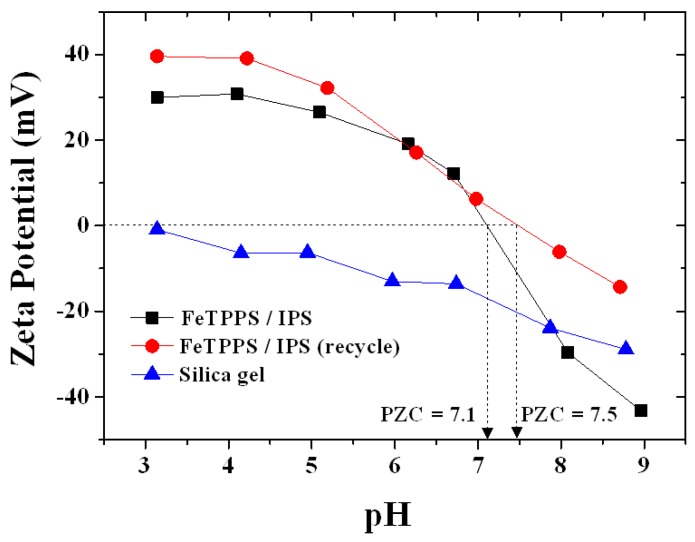
The pH dependence on the Zeta potential for silica, FeTPPS/IPS and the FeTPPS/IPS that was reused 5 times.

The change in the surface chemistry of the catalyst was characterized by zeta potential analysis, which is related to the surface charge (Figure 2). The unmodified silica had a negative zeta potential in the pH range of 3 to 9, which reflected a large negative surface charge due to the presence of deprotonated silanol groups. The FeTPPS/IPS catalyst had a negative zeta potential at pH values above 7.1. The FeTPPS/IPS catalyst had a positive zeta potential below pH 7.1, which can be attributed to the protonation of uncomplexed imidazole group in IPS. The zeta potential verse pH curve (● in Figure 2) for the reused catalyst was similar with fresh catalyst (■ in Figure 2). However, the magnitude of the zeta potential was increased in the pH range from 3 to 9, compared with the fresh catalyst. In addition, the point of zero charge (PZC) was shifted from pH 7.1 to 7.5 as a result of recycling. This may be due to the release and degradation of some FeTPPS during the oxidation reaction.

### 2.2. Influence of pH on the Degradation of TBBPA

**Figure 3:**
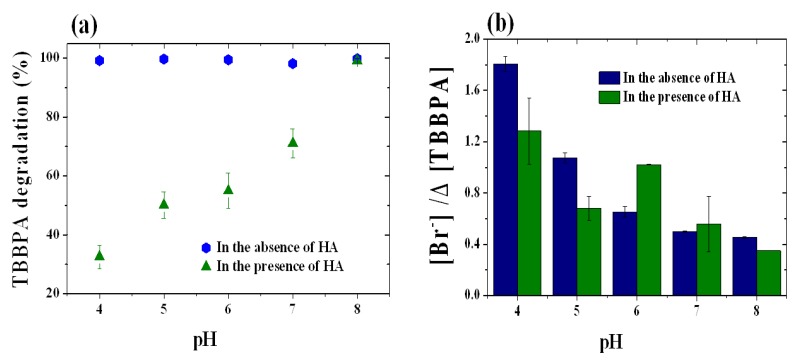
(**a**) Influence of pH on percentage TBBPA degradation (**b**) Influence of pH on debromination. The reaction conditions were as follow: [TBBPA]_0_, 50 μM; [FeTPPS/IPS], 10 μM (0.3 g L^−1^); [KHSO_5_], 1.0 mM; [HAs], 25 mg L^−1^; temperature, 25 °C; reaction time, 4 h.

Since the pH was not only related to the redox potential of the oxidant but also to species distribution of TBBPA and other concomitants in aqueous solutions, the influence of pH on the degradation of TBBPA was investigated. In the absence of HA, the degradation of TBBPA was not dependent on the pH of the solution. However, in the presence of HA, the reaction was clearly pH dependent, and the presence of HA also affected the degradation reaction. As shown in Figure 3a, in the presence of HA, the percentage of degraded TBBPA increased with increasing pH and the highest degradation performance was observed at pH 8, where more than 95% the TBBPA was degraded in the presence of HA, indicating that the oxidative degradation of TBBPA is inhibited by HA. This inhibition was enhanced in the lower pH range and became weaker at higher pH. The zeta potential of the FeTPPS/IPS indicated that the catalyst had negative surface charge at pH values above 7.1 and a positive surface charge at pH values below 7.1. Because HA has a large amount of negative surface charge [24], it can easily be adsorbed on the FeTPPS/IPS surface at a pH below 7.1. The interaction of TBBPA with catalytic sites could be blocked due to the adsorption of HA at a pH lower than 7. The surface charge of the catalyst changed to negative at pH values higher than 7.1. In this pH range, the HA appears to be excluded from the catalyst surface by electrostatic repulsion. Therefore, the inhibition by HA became weaker in a high pH range. Debromination was observed during the oxidation reaction in the pH range from pH 4 to 8 (Figure 3b). Although, in a previous study, no debromination was observed in the case of a homogeneous system [10], Br^−^ was clearly detected in the reaction mixture in the FeTPPS/IPS catalytic system. The low pH condition was beneficial for debromination, especially in the absence of HA, and the highest debromination value was found at pH 4. The highest rate of debromination was also observed at pH 4 in the presence of HA. However, compared with HA free conditions, the extent of debromination decreased in the presence of HA due to the drastic decrease in the rate of degradation of TBBPA. At pH 6 and 7, debromination was enhanced by HA, even the degradation of TBBPA was inhibited by HA. At pH 8, although the rate of debromination decreased slightly in the presence of HA, the percent TBBPA degradation was the highest in the pH range from 3 to 8 in the presence or absence of HA. In addition, the typical pH range for a leachate is reported to be 6.7–12 [2,3]. Therefore, the influence of HA and catalyst concentration on the degradation of TBBPA were examined at pH 8.

**Figure 4:**
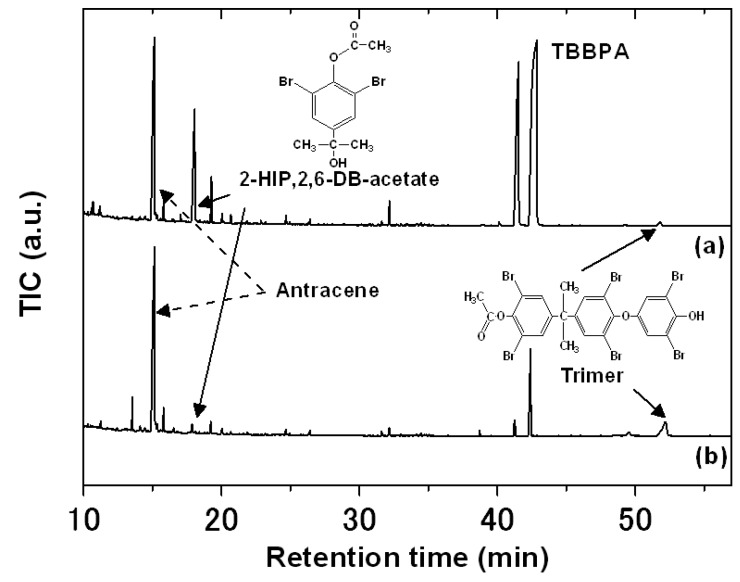
GC/MS chromatograms of *n*-hexane extract from the reaction mixture at pH 8 in the presence of HA. Reaction period (**a**) 1.5 h, (**b**) 5 h. Reaction conditions: [TBBPA]_0_, 50 μM; [FeTPPS/IPS], 10 μM (0.3 g L^−1^); [KHSO_5_], 1.0 mM; [HAs], 25 mg L^−1^, temperature 25 °C.

To identify the oxidation products produced in the reactions, *n*-hexane extracts of reaction mixtures were analyzed by GC/MS for the 1.5-h and 5-h reaction periods. Figure 4 shows one of the chromatograms for an *n*-hexane extract of reaction mixtures at pH 8 in the presence of HA. For the 1.5 h reaction period, the peak at 17.8 min of retention time was detected as a major oxidation product (Figure 4a). This peak was assigned as 4-(2-hydroxyisopropyl)-2,6-dibromophenol (2HIP-2,6DBP) acetate from the mass spectrum: *m*/*z* [relative intensity, fragment identify], 352 [2.65, M^+^], 310 [30.8, (M−CH_2_CO)^+^], 295 [100, (M−CH_3_CH_2_CO)^+^], 252 [48.3, C_6_H_4_OBr_2_^+^]. However, 2HIP-2,6DBP decreased for the 5 h reaction period, and the peak at 53.0 min of the retention time significantly increased (Figure 4b). This peak was assigned as the trimer of 2,6-dibromophenol and the mass spectral identification was as follows: *m*/*z* [relative intensity, fragment identify], 836 [71.0, M^+^], 794 [100, (M−CH_2_CO)^+^], 779 [44.2, (M−CH_3_CH_2_CO)^+^], 756 [48.3, (M−Br)^+^], 293 [14.8, C_6_H_2_(CH_3_CO_2_)Br_2_^+^], 267 [28.8, C_6_H_2_O(OH)Br_2_^+^]. The retention time and mass spectrum of 2HIP-2,6DBP acetate in the reaction mixtures were in good agreement with those for the acetate of the standard sample. In previous reports of TBBPA oxidation [5,6], while 2HIP-2,6DBP was found as one of the main byproducts, 2,6-dibromo-*p*-benzoquinone (2,6DBQ) was also detected as a main byproduct. However, no 2,6DBQ was found in the homogeneous FeTPPS-KHSO_5_ catalytic system [10], even at pH 4 and 6 as well as at pH 8 for any of the reaction periods. The patterns of oxidation products were also not varied by solution pH (for at pH 4 and 6) for the heterogeneous FeTPPS/IPS-KHSO_5_ catalytic system.

### 2.3. Influence of Catalyst Concentration on the TBBPA Degradation and Debromination

**Figure 5:**
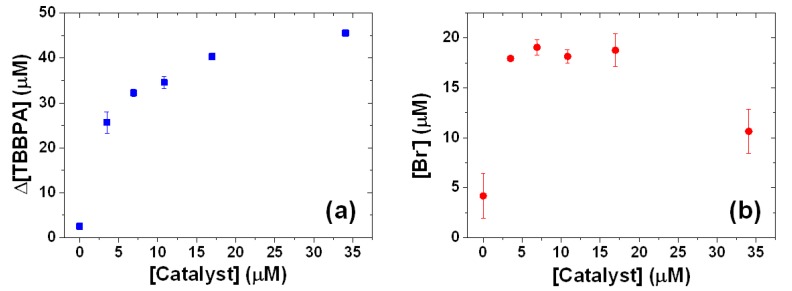
Influence of FeTPPS/IPS concentration on the (**a**) degradation and (**b**) debromination of TBBPA. [TBBPA]_0_, 50 μM; pH = 8; [KHSO_5_], 1 mM; temperature, 25 °C; reaction time, 35 min. The FeTPPS/IPS concentration at 0.3 g L^−1^ corresponds to 10 μM.

Figure 5 shows the influence of catalyst concentration on the degradation of and debromination of TBBPA, in which the Δ[TBBPA] represents the concentration of degraded TBBPA. A 0.7–3.4% decrease in the concentration of TBBPA was found in the presence of the FeTPPS/IPS (10–34 μM) without KHSO_5_. These results suggest that the contribution of TBBPA adsorption to the solid catalyst is minor in the case of Δ[TBBPA]. The Δ[TBBPA] steeply increased up to a concentration of 3.5 μM of the FeTPPS/IPS catalyst, and then gradually increased at concentrations up to 34 μM (Figure 5a). In the absence of the solid catalyst, a small amount of TBBPA degradation (3 μM) and Br^−^ release (4 μM) was observed for a 35 min reaction period. For the debromination (Figure 5b), the concentration of the released Br^−^ reached a plateau of 3.5–17 μM of the FeTPPS/IPS catalyst, but decreased at 34 μM. These results indicate that the presence of the catalyst enhances the degradation of TBBPA. The decrease in debromination at a FeTPPS/IPS concentration of 34 μM may be due to the enhanced oxidation of Br^−^ at higher catalyst concentrations. The turn over number for TBBPA degradation and debromination, as estimated for 3.5 μM of the FeTPPS/IPS catalyst, was 7.3 ± 0.3 and 5.1 ± 0.1, respectively.

### 2.4. Influence of HA Concentration

**Figure 6:**
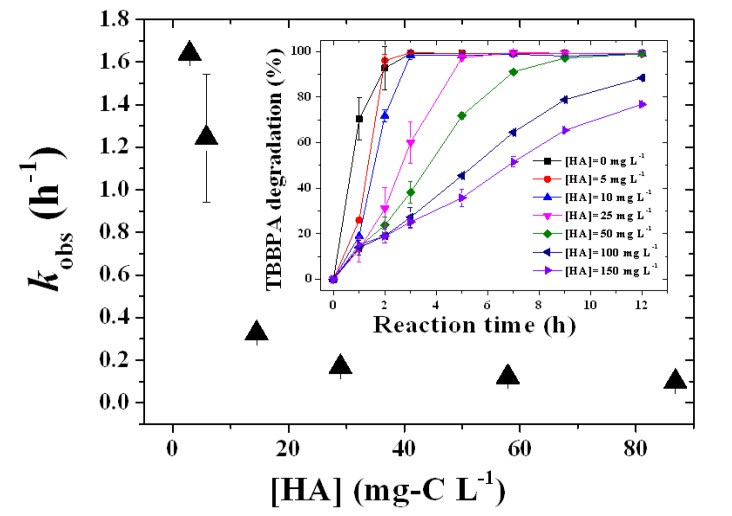
Influence of HA concentration on the pseudo-first-order rate constant (*k*_obs_) for TBBPA degradation and variations in the percent TBBPA degradation (insertion). The reaction conditions were as follow: [TBBPA]_0_, 50 μM; [FeTPPS/IPS], 10 μM (0.3 g L^−1^); [KHSO_5_], 1.0 mM; pH = 8; temperature, 25 °C.

HA is present at levels of 20–200 mg-C L^−1^ levels in landfill leachates [3], and HA can affect the distribution and oxidation reactions of organic pollutants. The influence of HA concentration was examined to assess the practical use of the FeTPPS/IPS catalyst. The pseudo-first-order rate constant (*k*_obs_) of TBBPA decreased with increasing concentration of HA. When the HA concentration increased from 2.8 to 14 mg-C L^−1^, the *k*_obs_ dramatically decreased from 1.6 to 0.3 h^−1^. With a further increase in the concentration of HA, the *k*_obs_ decreased further. From the insert in Figure 6, a drop-off in the initial degradation rate was observed with a small (2.8 mg-C L^−1^) mount of HA. However, when the reaction time was prolonged, the percent degradation TBBPA rapidly reached values higher than 95% within 5 h in the case of an HA concentration lower than 14 mg-C L^−1^. Over 95% the TBBPA was degraded within 9 h for HA concentrations of up to 29 mg-C L^−1^. Even in the presence of high concentrations of HA, 58–87 mg-C L^−1^, over 75% of the TBBPA was degraded within 12 h.

### 2.5. Reusability of FeTPPS/IPS

**Figure 7:**
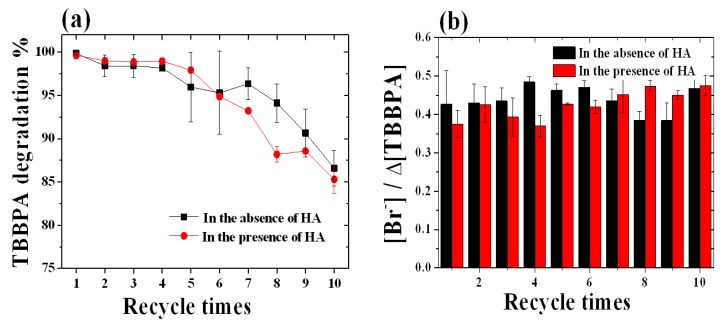
Reusability of the catalyst (**a**) TBBPA degradation, (**b**) number of bromide ions released. The reaction conditions were as follow: [TBBPA]_0_, 50 μM; [FeTPPS / IPS], 10 μM (0.3 g L^−1^); [KHSO_5_], 1.0 mM; [HAs], 25 mg L^−1^; temperature, 25 °C; pH = 8; reaction time, 4 h (in the absence of HA), 20 h (in the presence of HA).

In terms of using FeTPPS/IPS for water treatment, catalyst reusability is an important factor from the economical point of view. After each reaction, the catalyst was isolated on a filter, and then washed with deionized water and acetone. The catalyst had a high degree of durability as demonstrated by the recyclability test shown in Figure 7a. Over 95% of the TBBPA was degraded in the presence or absence of HA after five recyclings and more than 85% of the TBBPA was degraded after ten recyclings.

**Figure 8:**
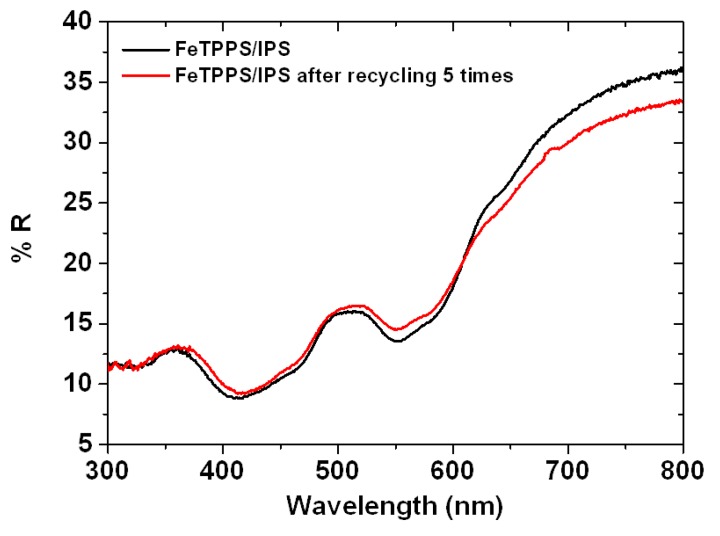
Diffuse reflectance UV-vis spectra for the FeTPPS/IPS catalyst before and after five recyclings.

The reused catalyst exhibited a good catalytic activity up to ten catalytic runs with only a small loss in degradation efficiency. The debromination was around 0.4 ([Br^−^]/Δ[TBBPA]) during the recyclability test (Figure 7b). However, the zeta potential of the FeTPPS/IPS increased slightly after five recyclings, as shown in Figure 2. At pH 8, the zeta potential of the reused catalyst was −6 mV and the fresh catalyst was −30 mV, indicating that the negative surface charge of the catalyst had decreased after the recyclability test. The HA would be predicted to be easily absorbed on the reused catalyst surface due to the change in surface charge, which would have an adverse impact on the degradation of TBBPA in the presence of HA. Therefore, with increasing catalyst reuse, the inhibition by HA became a larger issue (Figure 7a). The surface area of the reused catalyst (194 ± 10 m^2^ g^−1^) was similar to that for the fresh catalyst (215 ± 6 m^2^ g^−1^). In addition, Figure 8 shows Diffuse Reflectance UV-vis spectra for the fresh catalyst and after being used for five cycles. The fresh catalyst showed two peaks at 409 nm and 550 nm. After five recyclings, all of the peaks remained, indicating that the structure of the FeTPPS remained intact during the oxidative degradation reaction. These results show that the higher catalytic activity of FeTPPS/IPS catalyst was retained after several recyclings. 

## 3. Experimental

### 3.1. Materials

The soil humic acid (SHA) sample used in this study was extracted from Shinshinotsu peat soil, as described in a previous report [25]. Tetrabromobisphenol A (TBBPA), 3-isocyanatopropyltrimethoxysilane and *N*-(3-aminopropyl)imidazole, were purchased from Tokyo Chemical Industry (Tokyo, Japan). FeTPPS was synthesized according to the reported procedure [26]. KHSO_5_ was obtained as a triple salt, 2KHSO_5_·KHSO_4_·K_2_SO_4_ (Merck, Darmstadt, Germany).

### 3.2. Synthesis of Silica Supported FeTPPS Catalyst

**Scheme 1:**
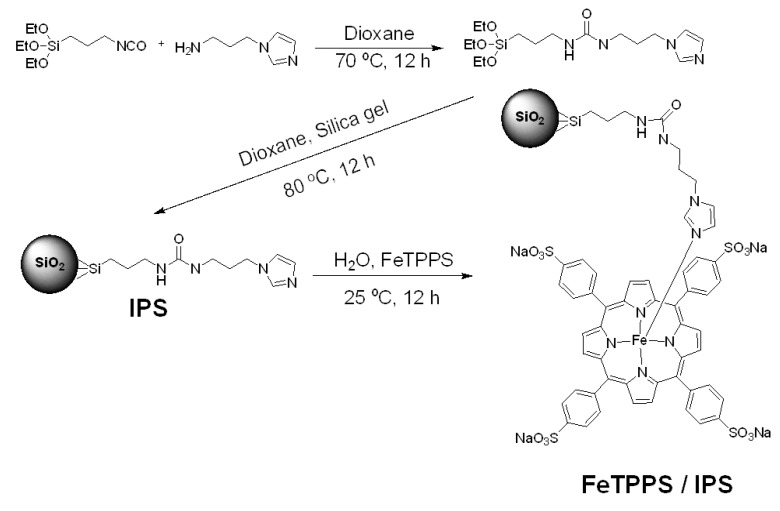
The synthesis of IPS and FeTPPS/IPS.

Scheme 1 shows the strategy used in the synthesis of the catalyst. The silica gel supported Fe(III)TPPS catalyst was synthesized by a previously reported method [18], with minor modifications. In a 2-neck flask, (3-isocyanatopropyl)triethoxysilane (1.3 mL) and *N*-(3-aminopropyl) imidazole (700 μL) were added to dioxane (20 mL) to synthesize 3-(1-imidazolyl)propylcarbamoyl-3′-aminopropyl-triethoxysilane. The mixture was stirred for 12 h at 70 °C. Subsequently, 1.5 g of silica gel (10–40 mesh, Wako Pure Chemicals, Osaka, Japan) was added, and the mixture was stirred at 80 °C for 12 h. The resulting solid was collected on a filter and consecutively washed with 0.5 M HCl, H_2_O, 0.1 M NaOH and finally washed with H_2_O. The 3-(1-imidazolyl)propylcarbamoyl-3′-aminopropylsilica (IPS) was then carefully dried overnight in vacuum oven at 50 °C. In a 100 mL flask, IPS (0.5 g) was added to FeTPPS solution (3.0 mM, 15 mL). The mixture was shaken at 25 °C, 150 rpm under 24 h in the dark. After the reaction, the FeTPPS/IPS was collected and washed with 1 M NaCl solution, ultra-pure water, and dried under vacuum.

### 3.3. Characterization of the Synthesized Catalyst

The catalyst loading amount was estimated using UV-visible absorption spectroscopy. UV-visible absorption spectroscopy and Diffuse Reflectance UV-vis spectra were obtained using a V-630 type spectrophotometer (Japan Spectroscopic Co. Ltd., Japan). FT-IR spectra were recorded using an FT/IR 600 type spectrometer (Japan Spectroscopic Co. Ltd.) with KBr pellets. The specific surface areas of the samples were obtained from N_2_ sorption isotherm at 77 K using a Beckman Coulter SA3100 (Brea, CA, USA). Zeta potentials were recorded using a Zetasizer Nano ZS90 (Malvern Instruments Ltd., Worcestershire, UK).

### 3.4. Assay for TBBPA Degradation

A 10 mL aliquot of a 0.02 M citrate/phosphate buffer at pH 4–8 was placed in a 100-mL Erlenmeyer flask. An aliquot (50 μL) of 0.01 M TBBPA in acetonitrile and the FeTPPS/IPS (3 mg) was then added to the buffer. Subsequently, aqueous solutions of 1,000 mg L^−1^ HA in 0.05 M NaOH solution and 0.1 M aqueous potassium monopersulfate (KHSO5, 100 μL) were added, and the flask was then allowed to shake at 25 °C in an incubator. After the reaction, the concentrations of the remained TBBPA were measured by an HPLC with a UV detector. The separation of TBBPA in the reaction mixture was accomplished with a COSMOSIL 5C_18_-AR-II column (4.6 mmø × 250 mm). The mobile phase consisted of a mixture of methanol and 0.08% of H_3_PO_4_ aqueous (78/22, v/v). The flow rate of the eluent and the detection wavelength were set to 1.0 mL min^−1^ and at 220 nm, respectively. The released Br^−^ was analyzed by ion chromatography (ICS-90 type, Dionex). The mobile phase was an aqueous mixture of 2.7 mM Na_2_CO_3_ and 0.3 mM NaHCO_3_, and the flow rate of the eluent was set at 1.5 mL min^−1^. The degradation percent of TBBPA was calculated by the following equation:

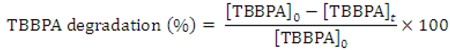

where [TBBPA]_0_ and [TBBPA]*_t_* represent the TBBPA concentrations remained in the reaction mixture before and after a *t*-h reaction period, respectively. The pseudo first-order reaction rate constant, *k*_obs_ (h^−1^), was estimated by non-linear least square regression analysis of the dataset for reaction time (h) and [TBBPA]*_t_*/[TBBPA]_0_ to below equation:

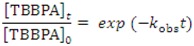



The turnover number for TBBPA degradation and debromination was calculated by dividing the concentration of degraded TBBPA (Δ[TBBPA] = [TBBPA]_0_ − [TBBPA]*_t_*) or released Br^−^ by the catalyst concentration.

For the analysis of oxidation products, 1 M aqueous ascorbic acid (1 mL) was added, and pH of the solution was adjusted to 11–11.5 by adding aqueous K_2_CO_3_ (600 g L^−1^). Subsequently, acetic anhydride (5 mL) was added dropwise to the solution, and a 1 mM anthracene solution in hexane (0.5 mL) was added as an internal standard (ISTD) for the GC/MS analysis. This mixture was doubly extracted with *n*-hexane (10 mL), and the extract was then dried over anhydrous Na_2_SO_4_. After filtration, the extract was evaporated under a stream of dry N_2_, and the residue was dissolved in *n*-hexane (0.25 mL). An aliquot of the extract (1 μL) was introduced into a GC-17A/QP5050 GC/MS system (Shimadzu, Kyoto, Japan). A Quadrex methyl silicon capillary column (0.25 mm id × 25 m) was employed in the separation. The temperature ramp was as follows: 65 °C for 1.5 min, 65–120 °C at 35 °C min^−1^, 120–300 °C at 4 °C min^−1^ and a 300 °C held for 10 min.

## 4. Conclusions

A FeTPPS/IPS catalyst was synthesized and its use in the degradation and debromination of TBBPA in the absence and presence of HA, a major component of leachates, was examined. This catalytic system was pH independent in the absence of HA and the highest catalytic activity was found to be at pH 8 in the presence of HA. Although the presence of HA retarded the degradation of TBBPA, over 95% of the TBBPA was degraded in the case of HA 28 mg-C L^−1^. In addition, FeTPPS/IPS exhibited good catalytic activity for up to ten recyclings. As a green and efficient catalyst, FeTPPS/IPS has promise for use in the field of pollution control.
